# Shikonin-enhanced cell immunogenicity of tumor vaccine is mediated by the differential effects of DAMP components

**DOI:** 10.1186/s12943-015-0435-9

**Published:** 2015-09-24

**Authors:** Tien-Jen Lin, Hsin-Ting Lin, Wei-Ting Chang, Pradeep Mitapalli. S, Pei-Wen Hsiao, Shu-Yi Yin, Ning-Sun Yang

**Affiliations:** Graduate Institute of Injury Prevention and Control, Taipei Medical University, Taipei, Taiwan; Department of Neurosurgery, Taipei Medical University - Wan Fang Hospital, Taipei, Taiwan; Agricultural Biotechnology Research Center, Academia Sinica, Taipei, Taiwan; Graduate Institute of Biotechnology, National Chung Hsing University, Taichung, Taiwan; Taiwan International Graduate Program (TIGP), Molecular and Biological Agricultural Sciences Program, Academia Sinica, Taipei, Taiwan; Room 641, Agricultural Biotechnology Research Center, Academia Sinica, No. 128, Sec. 2, Academia Road, Nankang, Taipei, Taiwan

**Keywords:** Anti-metastasis, Shikonin, Immunogenic cells death, Dendritic cell vaccine

## Abstract

**Background:**

The tumor cell lysate-pulsed, dendritic cell (DC)-based cancer vaccine approaches are being actively evaluated for application to cancer immunotherapy, hopefully at a personalized medicine base. There is apparently an emerging technical problem however, the lack of highly efficacious potency in activation of patient’s DCs for T-cell priming and the associated process for presenting tumor immunogenicity.

**Methods:**

One strategy to address this is to consider the manipulation of the tumor immunogenic cells death (ICD) complex ex-vivo for maximal activation of DC efficacy. In our previous study we showed that phytochemical shikonin (SK) can drastically enhance ICD activity in mouse tumor cells treated ex-vivo, and the resultant tumor cell lysate (TCL) can effectively augment such SK-TCL pulsed DC vaccine activity in vivo in anti-tumor activities. In this study, we investigated the specifics and the multi-functional effects of various damaged associated molecular pattern (DAMP) components of the ICD complex for their participation, roles and potential cross talks in activating DCs, as measured by five different functional assays.

**Results:**

Among three DAMPs tested, HSP70 and CRT mediate a key role in SK-TCL-induced DC immunity for both CD4^+^ and CD8^+^ T cell proliferations *in vitro.* HSP70 is the most important component, followed by CRT, then HMGB1 in facilitating DC immunity on suppressing metastasis of mouse 4 T1 mammary tumors and prolonging survival in test mice. Only HSP70, but not CRT or HMGB1, is effective for the suppression of both granulocytic and monocytic MDSC populations *in vivo*. Both HSP70 and HMGB1, but not CRT, are essential in activating the expression of three key ICD molecules-associated receptors on test DCs. Each of the three test ICD proteins can exhibit a distinguishable pattern in stimulating the expression of four key chemokines in test DCs.

**Conclusion:**

Our findings on the differential roles or effect of various ICD components in activating vaccinated DCs may help formulate new strategies for future cancer vaccine designs.

## Introduction

Simultaneous changes of damage-associated molecular patterns (DAMPs) associated with immunogenicity, including glucose-related protein (GRP), heat shock proteins (HSP), calreticulin (CRT), high mobility group box 1 (HMGB1) and others are well characterized in mechanisms for immunogenic cell death (ICD) [1]. DAMPs are found within living cells as a group of natural endogenous adjuvants, and in response to danger signals, they become stimulatory to the adaptive immune system [2, 3]. Priming of an antigen-specific effector T cell response and a strong T cell immunity can come after the activation of a series of receptors expressed by dendritic cells (DCs). This same process also can enable the progression of antigen uptake and generate an array of molecular activities associated with the activation of DCs.

Toll-like receptor 4 (TLR4) is essential for sensing DAMPs, as well as the resultant activation of DCs and the induction of Th1 response [4, 5]. DAMPs that serve as agonists of TLR4 include heat shock proteins (e.g., HSP60, HSP70, HSP90, HSPB8, and endoplasmin), HMGB1 and uric acid crystals [4, 5]. Heat shock proteins have attracted increasing attention due to their ability to chaperone a wide array of peptides generated in dying cells [6, 7]. In addition, treatment with chemotherapeutic agents such as anthracyclines and bortezomib is known to induce a pre-mortem stress response that exerts immunostimulatory effects after the exposure of heat shock proteins such as HSP70 and HSP90 on the surface of dying cancer cells [5]. Some conventional chemotherapeutics (e.g., the nucleoside analog gemcitabine) as well as targeted agents [e.g., the epidermal growth factor receptor (EGFR) inhibitor, erlotinib] result in increased levels of class I or class II MHC molecules on the tumor cell surface, effectively facilitating their recognition by the immune system [6, 7]. Chemotherapy may thus stimulate the immunogenicity of cancer cells at multiple levels, and contribute to the induction of efficient anticancer immune responses [6, 7]. In particular, HSP-peptide complexes isolated from tumor cells, or reconstituted by covalent cross-link or fusion-protein strategies are critical for the loading of MHC-I with epitopes, triggering effective tumor-specific T-cell responses and antitumor effects via cross-presentation by DCs [6, 7].

Shikonin (SK) and its derivatives are the primary active components isolated from the root tissues of a traditional Chinese medicinal herb, *Lithospermum erythrorhizon* and are well-studied bioactive phytochemicals [8]. We previously found that shikonin can effectively induce the expression of specific DAMPs, which in turn activated a caspase cascade in treated tumor cells [8]. We further showed that in combination with pathogen-associated molecular patterns (PAMPs), such as toll-like receptors, shikonin-induced tumor cells lysate (SK-TCL) can activate DCs to phenotypic and functional maturation, which in turn increased the cytotoxic T lymphocyte activity contributing to efficacious retardation of tumor growth and prolonged the survival of test mice [8]. Our findings suggest that shikonin may serve as an adjuvant for use in TCL-loaded DC vaccines against cancer or other immunotherapeutic applications. However, the exact mechanisms, signaling pathways and regulatory apoptotic molecules that are involved in the process of immunogenic cell death induced by shikonin are still unknown.

In this study, we evaluated the role of the key components of DAMPs in mediating the interaction between tumor lysate and treated DCs, and the mechanism by which anti-tumor immunity is induced by DCs pulsed with shikonin-treated TCLs. We further analyzed the individual involvement of mammary tumor cell-derived ICD constituents (i.e., HSP70, CRT and HMGB1) in the promotion of the anti-metastatic activity of SK-TCL pulsed DCs. Within the same context, because doxorubicin (Dox) has been shown to act as a highly effective immunogenic cell death inducer [8], both Dox- and SK-treated TCLs were analyzed to evaluate the key molecular signals, which may involve a series of receptors expressed by DCs for stimulating the presentation of tumor antigens to T cells. We believe these findings provide important and specific evidence for use of shikonin in cancer immunotherapy, and in the future they may be useful to aid the development of tumor associated antigen (TAA)-based DC vaccines.

## Materials and methods

### Compounds and antibodies

Shikonin (SK) was purchased from Tokyo Chemical Industry (Tokyo, Japan), and doxorubicin (Dox) was from Sigma (St. Louis, MO, USA). The three antibodies used for depletion of specific DAMP proteins in tumor cell lysate (TCL) were anti-HSP70 (rabbit plyoclonal; GeneTex), anti-CRT (rabbit polyclonal; Abcam) and anti-HMGB1 (rabbit plyoclonal; GeneTex). The same antibodies and anti-β-actin antibody (rabbit polyclonal; Abcam) were also used as primary antibodies for western blot analyses. HRP-conjugated secondary antibody (goat polyclonal; Abcam) was used as a secondary antibody.

### Cell lines

Mouse mammary carcinoma cell lines 4 T1 and 4 T1-luc2 (i.e., 4 T1 cells transfected by a firefly luciferase cDNA expression vector [9] were kindly provided as a gift by Dr. Hsiao (ABRC, Academia Sinica, Taipei). Transgenic 4 T1-luc2 cells were employed in the spontaneous metastasis experimental model after surgical resection of the primary tumor. The evaluation of bioluminescence signals from implanted 4 T1-luc2 tumor cells in test mice was performed by using a non-invasive *in vivo* imaging system (IVIS) (Calipers, Hopkinton, MA). Both 4 T1 and 4 T1-luc2 cells were maintained in RPMI-1640 complete medium (i.e., RPMI-1640 supplemented with 10 % FBS, 100 μM non-essential amino acids, 100 μM sodium pyruvate, 100 μg/ml streptomycin and 100 unit/ml penicillin) and grown in a 5 % CO_2_ incubator at 37 °C.

### Preparation of tumor cell lysates

The 4 T1 tumor cell lysate (TCL) samples were prepared as described previously [9]. Briefly, at 50 % confluence, 4 T1 cells were treated with shikonin (SK) or doxorubicin (Dox) at 5 μM for 24 h for induction of immunogenic cell death (ICD). SK- or Dox-treated 4 T1 cells were then collected and resuspended in PBS, frozen in liquid nitrogen for 1.5 min and thawed for another 4 min at 4 °C by sonication. The freeze–thaw cycles were repeated four times. After the final thaw, TCL suspensions were centrifuged at 12,000 rpm for 30 min, and the supernatant was used as the source of tumor antigens. Tumor cell lysates were frozen at −80 °C until use.

### Antibody-mediated protein depletion for SK-TCLs

The Dynabeads Antibody Coupling Kit (Life Technologies; 14311D) was used to pull down individual intracellular ICD-related protein molecules in 4 T1 cells according to the manufacturer’s recommendations, yielding 10 mg antibody-coupled beads. After the antibody coupling reaction, aliquots of 500 μg SK-TCL were reacted with 2 mg antibody-coupled beads on a roller shaker at room temperature for 1 hour. The SK-TCL samples were then placed on a magnet for 1 min and the beads were collected along the tube wall. Different preparations of SK-TCL supernatants, namely [SK-TCL(−HSP70), SK-TCL(−CRT) and SK-TCL(−HMGB1)] were collected and used for DC activation experiments.

### Mice

Groups of female BALB/c mice aged 6–8 weeks were purchased from the National Laboratory Animal Breeding and Research Center, Taipei, Taiwan. Experimental mice were maintained in a standardized laminar airflow cabinet under specific pathogen-free conditions, and all manipulation and experimental protocols involving animals were approved by the IACUC office of Academia Sinica, Taipei.

### Mouse bone marrow-derived dendritic cells

Mouse bone marrow-derived dendritic cells (BMDCs) were generated and modified as previously described [10]. Briefly, mouse bone marrow tissues were collected from BALB/c mice, and erythrocytes were removed. The derived bone marrow cells were cultured in 30 ml complete RPMI 1640 medium (see above) supplemented with 20 ng/mL GM-CSF and 50 μM 2-mercaptoethanol. Two days later, two-thirds of the original medium was replaced by 30 mL fresh medium. On day 5, the floating cells were gently removed and the culture replenished with fresh medium containing 20 ng/mL GM-CSF and 20 ng/mL IL-4. On day 7, the non-adherent and loosely adherent DCs in culture were harvested and used as the source of dendritic cells for various vaccines. DCs were routinely generated in this manner, and found to be mainly immature DCs with 85 % of cells expressing CD11c^+^, and displaying the typical morphological features of DCs.

### Dendritic cell activation

The procedure for performing TCL-mediated activation of BMDCs was performed as previously described [10]. Briefly, BMDCs were incubated for 2 h with various TCL samples containing 200 μg protein/ml. LPS at 1 μg/ml was added to the medium for co-cultivation with TCL-loaded DCs for another 22 h. DC samples treated with 1 μg/ml LPS only for 24 h (mDCs) were used as the vehicle control for TCL stimulation. DC samples reacted with naive-TCLs (i.e., cell lysates collected from 4 T1 tumor cells that were treated without shikonin or doxorubicin stimulation) were designated as mDCs + Naive-TCL. DC samples treated with TCLs obtained from 4 T1 cells treated for 24 h with shikonin or doxorubicin stimulation were designated as mDCs + SK-TCL and mDCs + Dox-TCL, respectively. Similarly, DCs reacted with SK-TCL samples with which the specific ICD proteins were depleted with antibodies against HSP70, CRT or HMGB1 were designated as mDCs + SK-TCL(−HSP70), mDCs + SK-TCL(−CRT) and mDCs + SK-TCL(−HMGB1), respectively. These test DCs were then compared for their activity in stimulating T-cell proliferation by mixed lymphocyte reaction (MLR) assay, and for their anti-metastatic effect on 4 T1 tumors *in vivo*. The conditional culture media of test DCs were also collected for cytokine array analysis.

### Mixed lymphocyte reaction assay

Mouse CD4^+^ and CD8^+^ T cells were isolated by magnetic activated cell sorting (MACS) selection of splenocytes with anti-CD4 or anti-CD8 antibody-coated microbeads (Miltenyi Biotech, Bergisch Gladbach, NRW), resulting in >98 % purity and >98 % viability in general. Co-cultivation of the DC-T cell system was performed as previously described [10, 8]. Briefly, aliquots of 1 × 10^5^ CD4^+^ or CD8^+^ T cells were co-cultured with BMDCs (at a DC/T ratio of 1:10) for 4 days in a final volume of 200 μl medium, and the BMDC-induced T-cell proliferation activity was determined by Cell Proliferation ELISA (Roche Applied Science, Indianapolis, IN).

### 4 T1 mammary carcinoma-tumor resection model

Mice were injected subcutaneously with 4 T1-Luc2 cells (5 × 10^5^/50 μl PBS/mouse) into the fourth mammary fat pad under isoflurane anesthesia. Tumor growth was monitored by measuring the tumor volume, as length × (width)^2^/2. After tumors were established (180–200 mm^3^) on day 15, test mice were divided into different groups (8 mice/group) and the primary tumors were surgically resected. For immunization with DC vaccines, mice were subjected to control treatment (PBS) or with specific DC vaccines (1 × 10^6^/200 μl PBS/mouse) via intravenous injection at 0, 7 and 14 days post tumor resection. To monitor the progression of metastatic tumors, bioluminescence signals from the 4 T1-luc2 tumor cells in test mice were analyzed using a non-invasive IVIS imaging system (Calipers, Hopkinton, MA) after intraperitoneal injection of 150 mg/kg D-luciferin (NanoLight Technology, Pinetop, AZ). For detection of myeloid-derived suppressor cells **(**MDSCs), peripheral blood cells from test mice were collected at 10 days post the secondary boosting of DC vaccine or PBS (control). The criteria used to determine humane endpoint was strictly based on the Guidelines for Determining Endpoints and Humane Termination of Animals provided by the Institutional Animal Care and Use Committee (IACUC) of Academia Sinica, Taiwan. All test mice were euthanatized by CO2 inhalation at the end of experiments.

### Flow cytometry analysis of surface and intracellular markers of regulatory T cells and myeloid-derived suppressor cells

For detection of myeloid-derived suppressor cells **(**MDSCs), peripheral blood cells from DC vaccine-immunized and control mice were collected, and leukocytes were stained for 30 min at 4 °C with antibodies against specific cell markers, including FITC-conjugated anti-mouse CD11b (for cell surface), APC-Cy7-conjugated anti-mouse Ly6C and PE-conjugated anti-mouse Ly6G, (both for intracellular staining). All three antibodies were obtained from Biolegend, (San Diego, CA). The percentages of monocytic and granulocytic MDSCs were gated on CD11b^+^Ly-6C^+^ cells and CD11b^+^Ly-6G^+^ cells, respectively [11], followed by cell permeabilization with the Cytofix/Cytoperm Plus kit according to the manufacturer’s protocol for test antibodies and the reagent kit from BD Pharmingen (San Diego, CA). Fluorescence signals were detected by flow cytometry.

### Cytokine array analysis

BMDCs (5 × 10^6^/6 ml) were individually incubated with different TCL samples at 200 μg protein/ml for 2 h and then treated with LPS (1 μg/ml) for another 22 h. Conditioned culture media of test DCs were collected and analyzed by Mouse Cytokine Array Panel A (R&D Systems, Minneapolis, MN). A total of 40 cytokines/chemokines were analyzed in this assay, which was performed according to the manufacturer’s protocol. Briefly, aliquots of 15 μl cytokine array detection antibody cocktail were added to each test sample and incubated at room temperature for one hour. Cytokine array membranes were then incubated with sample/antibody mixtures overnight at 4 °C on a shaker. Test samples on membranes were washed 3 times with 1× wash buffer, each for 10 min. Membranes were then incubated with Streptavidin-HRP solution for 30 min at room temperature on a shaker. Membranes were washed 3 times again as before. Aliquot of 1 ml Chemi Reagent Mix was added onto each membrane and incubated for 1 min. The membrane was drained and exposed to X-ray film for 1–20 min. Data were analyzed using Alpha View SA 3.4 software. Mean values of spotted duplicates were calculated and normalized with the mean values of reference spots (i.e., internal positive controls). Cytokines/chemokine expression levels were indicated as fold-change when compared to those of the control group (mDCs + Naive-TCL).

### Western blot assay

Western blot procedures were performed as previously reported [8]. Tumor cell lysate samples were prepared as previously described [8, 10]. The 4 T1 TCL protein samples were resolved by SDS-PAGE using 8, 10 or 15 % stepwise gels. The resolved proteins were transferred to a PVDF membrane (Novex, San Diego, CA, USA), and the membrane was blocked with 5 % non-fat dry milk in PBST buffer [phosphate-buffered saline (PBS) containing 0.1 % Tween 20] for 60 min at room temperature. The membranes were incubated with primary antibodies (1:1000 dilution) overnight at 4 °C, then with HRP-conjugated secondary antibody (1:100,000 dilution) for 1 h at room temperature, and washed with PBST buffer. The transferred proteins were visualized with an enhanced chemiluminescence (ECL) detection assay kit (Amersham Pharmacia Biotech, Buckinghamshire, UK). Quantification of bands was performed using Image J software.

### Statistical analysis

Statistical analysis was performed using an unpaired, two-tailed Student’s t-test. Statistical analyses were conducted with GraphPad Prism 5.0 (San Diego, CA). Differences in tumor metastasis mouse survival rate were determined by a log-rank (Mantel-Cox) test of the Kaplan-Meier survival curves. All statistical tests were two-sided. A P-value of less than 0.05 was considered significant (*, *P* < 0.05; **, *P* < 0.01; ***, *P* < 0.001; n.s, no significance).

## Results

### Effect of shikonin-treated 4 T1 tumor cell lysates (SK-TCL) on DC-activated T-cell proliferation

**Fig. 1:**
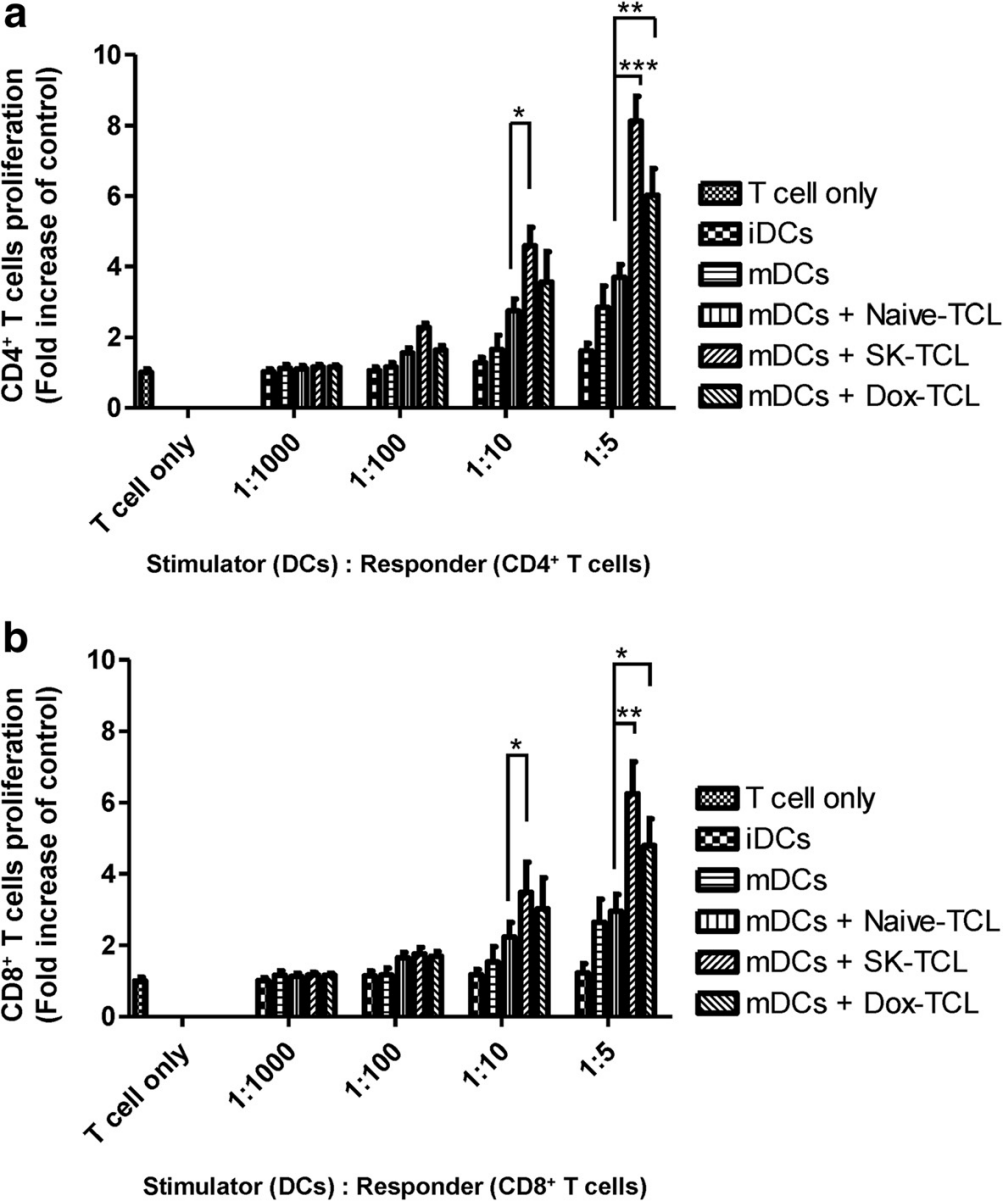
Shikonin-treated 4 T1 tumor cell lysate effectively activates DCs that in turn induce T-cell proliferation *in vitro*. SK-TCL or Dox-TCL samples were prepared from transgenic Luc2-labelled 4 T1 cells that were treated with shikinon or doxorubicin at 5 μM for 24 h. Mouse bone marrow-derived DCs were then treated with Naive-, SK- or Dox-TCL samples and used as stimulator cells. Splenic CD4^+^ and CD8^+^ T cells were collected from syngeneic mice and employed as responder cells. Proliferation activities of **a** CD4^+^ and **b** CD8^+^ T cells were analyzed by mixed lymphocyte reaction (MLR) assay. Ratios of stimulator to responder cells were set between 1:1000 and 1:5. T-cell proliferation activity is represented as fold change over the control (T cells only). Data represent the mean ± SD of three biological replicates, and an independent experiment showed similar results

To evaluate the efficacy of using shikonin-induced immunogenic cell death (ICD) in tumor cells to enhance DC-mediated T-cell proliferation activity, test DCs were stimulated in culture with shikonin-treated 4 T1 tumor cell lysates (SK-TCL), and the effect of the resultant DCs on T-cell proliferation was tested by MLR assay. As compared to treatment with mDCs only, all TCL-treated mDC groups, including Naive-TCL, SK-TCL and Dox-TCL, were found to stimulate dose-dependent proliferation of both CD4^+^ and CD8^+^ cells (Fig. 1a and 1b, respectively) at a DC/T cell ratio between 1:1000 and 1:5. At the ratio of 1:5, when compared to mDC group, the SK-TCL group was found to significantly induce CD4^+^ and CD8^+^ T-cell proliferation up to 5.27- and 3.53-fold (Fig. 1a and 1b), respectively, as calculated by the formula [(SK-TCL/iDC)-(mDC/iDC)]/[(Naive-TCL/iDC)-(mDC/iDC)]. In comparison, Dox-TCL exhibited only a 3.47- and 2.40-fold change in CD4^+^ and CD8^+^ T-cell proliferation activities, as shown in Fig. 1a and 1b, respectively. These results suggest that SK-TCL-activated mDCs can effectively induce the proliferation of both CD4^+^ and CD8^+^ T cells. These activities were not only significantly higher than the Naive-TCL group, they were even moderately higher than those obtained from the Dox-TCL group.

### SK-TCL-activated DCs effectively suppress the metastasis of 4 T1 cells in an orthotopic tumor resection model *in vivo*

**Fig. 2:**
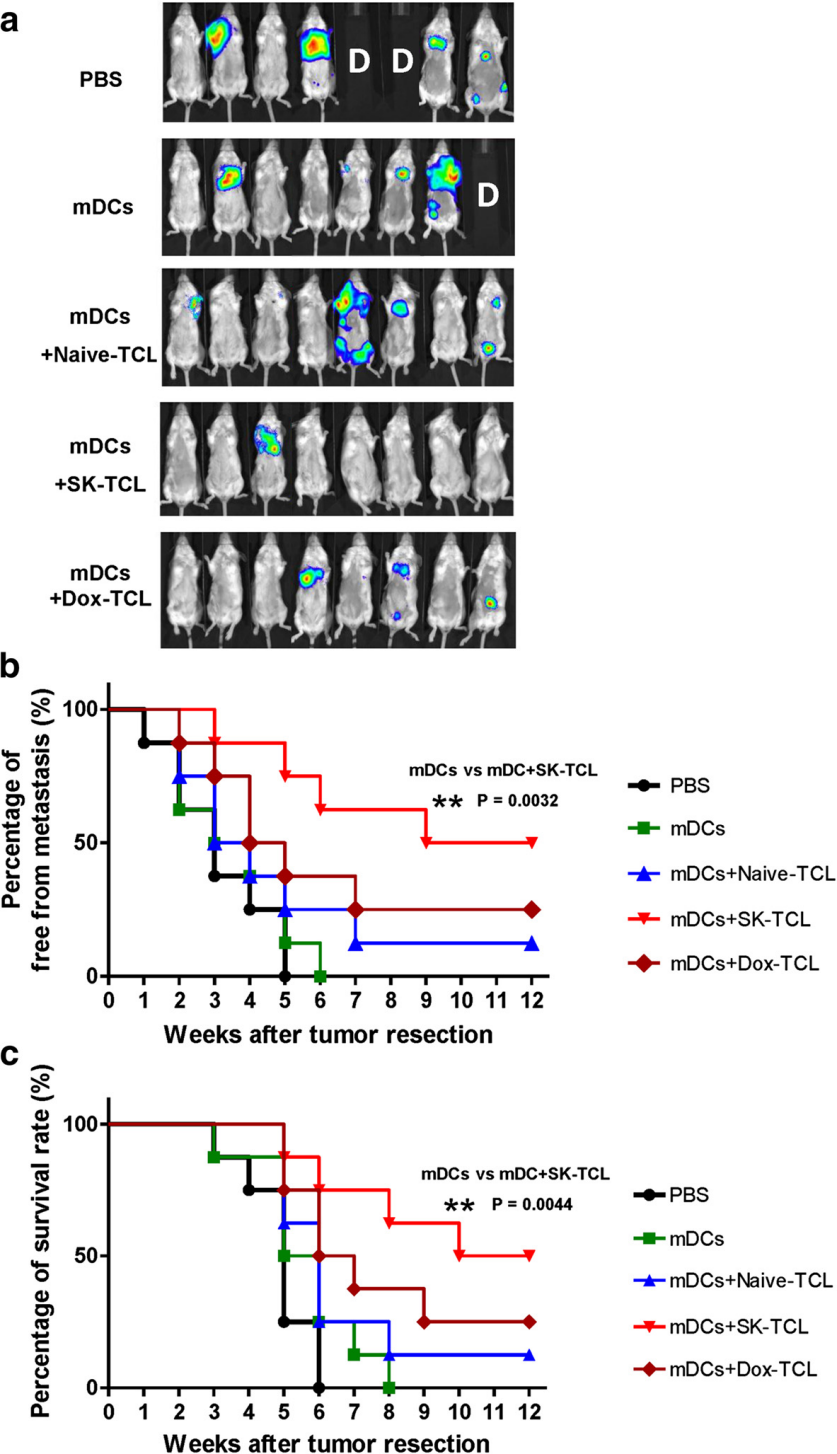
Adjuvant effect of SK-TCL (formulation) used with a DC vaccine, in 4 T1 tumor metastasis in mice. Test mice were injected subcutaneously with 4 T1-Luc2 cells (5 × 10^5^ cells/100 μl PBS/mouse) into the mammary fat pad under isoflurane anesthesia. At 15 days post tumor cell implantation, primary tumors were surgically resected. Specifically formulated DC vaccines (1 × 10^6^ DCs/200 μl PBS/mouse) were delivered via tail vain injection to mice at 0, 7 and 14 days post tumor resection. **a** Representative *in vivo* bioluminescent images of test mice (8 mice/group) treated with PBS, mDCs, mDCs + Naive-TCL, mDCs + SK-TCL and mDCs + Dox-TCL vaccines after resection of the 4 T1 orthotopic primary tumors. The red signals represent the highest level on the colorimetric scale. **b** Percentage of whole body (all organs) free from metastasis in mice (8 mice/group). The metastasis levels of tumors in test mice were scored within the indicated time course as revealed by bioluminescence imaging. **c** Survival rate of test mice after resection of 4 T1 tumors and treatment with the indicated DC vaccine regimes. Similar trends of results were obtained from four independent experiments

The results outlined above and shown in Fig. 1 suggest that SK-TCL may possess potential for augmenting T cell-mediated anti-tumor activity. We therefore next evaluated the efficacy of use of Naive-TCL, SK-TCL or Dox-TCL as adjuvants for a DC vaccine formulation aiming to inhibit tumor metastasis in a mouse 4 T1 mammary tumor-resection model. A bioluminescent, transgenic luciferase-labeled 4 T1 mouse mammary carcinoma cell line (a gift provided and developed by Dr. P.W. Hsiao of Academia Sinica, Taiwan) was used as a tumor metastasis model in BALB/c mice. At days 0, 7 and 14 post tumor resection, test mice were injected with different vaccine regimes via tail vein injection. Bioluminescent imaging analysis of mouse tumors was monitored and scored using a non-invasive *in vivo* imaging system (IVIS). Among all the test groups, the SK-TCL vaccine formulation resulted in the highest and most significant inhibition of tumor metastasis, as seen with the lowest level of luminescent imaging of tumor foci (Fig. 2a). When compared with the mDC (control) group, SK-TCL also showed the strongest anti-tumor metastasis activity, as scored by the percentage of mice free from metastasis (Fig. 2b). Fig. 2a and 2b show that more than 50 % of mice in the mDC group suffered tumor metastasis in the first month after tumor resection. In contrast, close to 85 % of mice in the SK-TCL group showed no detectable tumor metastasis. Furthermore, SK-TCL-activated DCs also significantly prolonged the survival time of test mice as compared to the mDC group mice (Fig. 2c). Together, our findings suggest that the use of SK-TCLs to treat DCs, as a vaccine formulation strategy, can effectively suppress 4 T1 tumor metastasis and prolong the life span of test mice.

### Shikonin effectively increases the expression of HSP70 and CRT, but not HMGB1, in 4 T1 cells

**Fig. 3:**
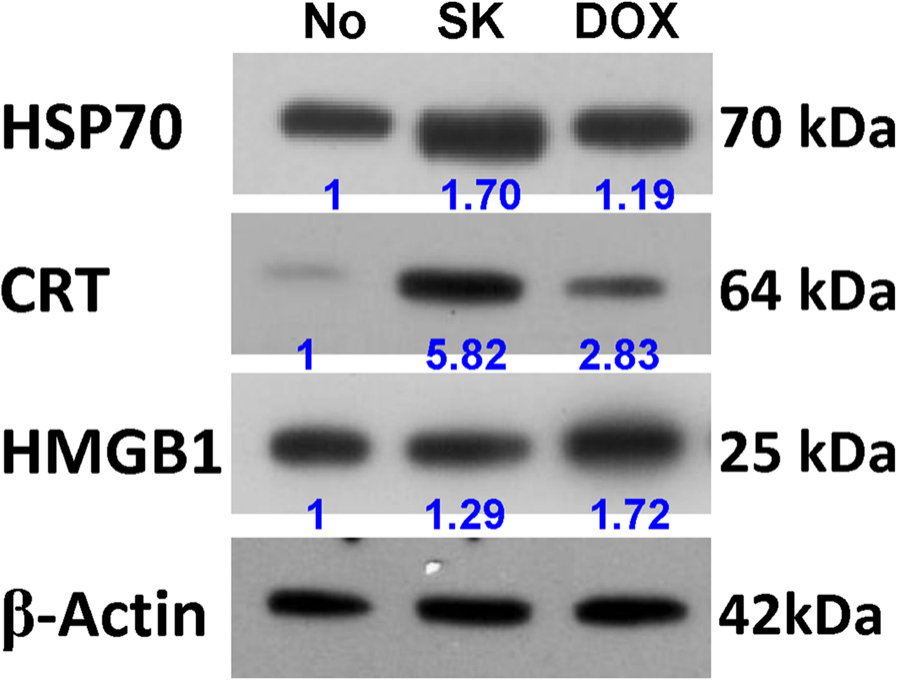
Western blot analyses for expression of HSP70, CRT and HMGB1 in 4 T1 cells. Test 4 T1 cells dispensed in 6-well plates (3 × 10^5^ cells/well) were incubated with vehicle, SK or Dox at 5 μg/ml for 24 h. Beta-actin was used as a loading control. Three independent experiments showed similar patterns as the data shown here

Certain damage-associated molecular patterns (DAMPs) are known to play an important role in activating immune cell-mediated immunogenicity of cancer cells [12, 2, 13]. Furthermore, our results shown in Fig. 2 suggest that shikonin (SK) can effectively induce tumor cell ICD, and this effect constitutes good adjuvant activity for a DC-based cancer vaccine. To further evaluate the possible specific roles of the DAMP components in the ICD-associated activities of shikonin-treated tumor cells, we next compared the changes in expression of major ICD components, HSP70, CRT and HMGB1, in 4 T1 cells in response to SK treatment. In this experiment, doxorubicin (Dox), which is known to stimulate ICD [14], was used a positive control. Treatment with SK at 5 μM for 24 h significantly increased the expression of HSP70 and CRT on mouse 4 T1 mammary tumor cells (Fig. 3), but had little or no effect on HMGB1 expression (Fig. 3) or other ICD markers tested (e.g., HSP90) (data not shown). In comparison, Dox treatment substantially increased the expression of CRT and HMGB1, but had limited effect on HSP70 expression. These data suggest that SK and Dox exert a differential effect on the expression profile of different ICD component markers, and this difference may contribute to different mechanisms of ICD and the resultant adjuvant effect on DC vaccines (see below).

### HSP70 and CRT are important DAMP molecules for the SK-TCL-mediated DC activation of T-cell proliferation

**Fig. 4:**
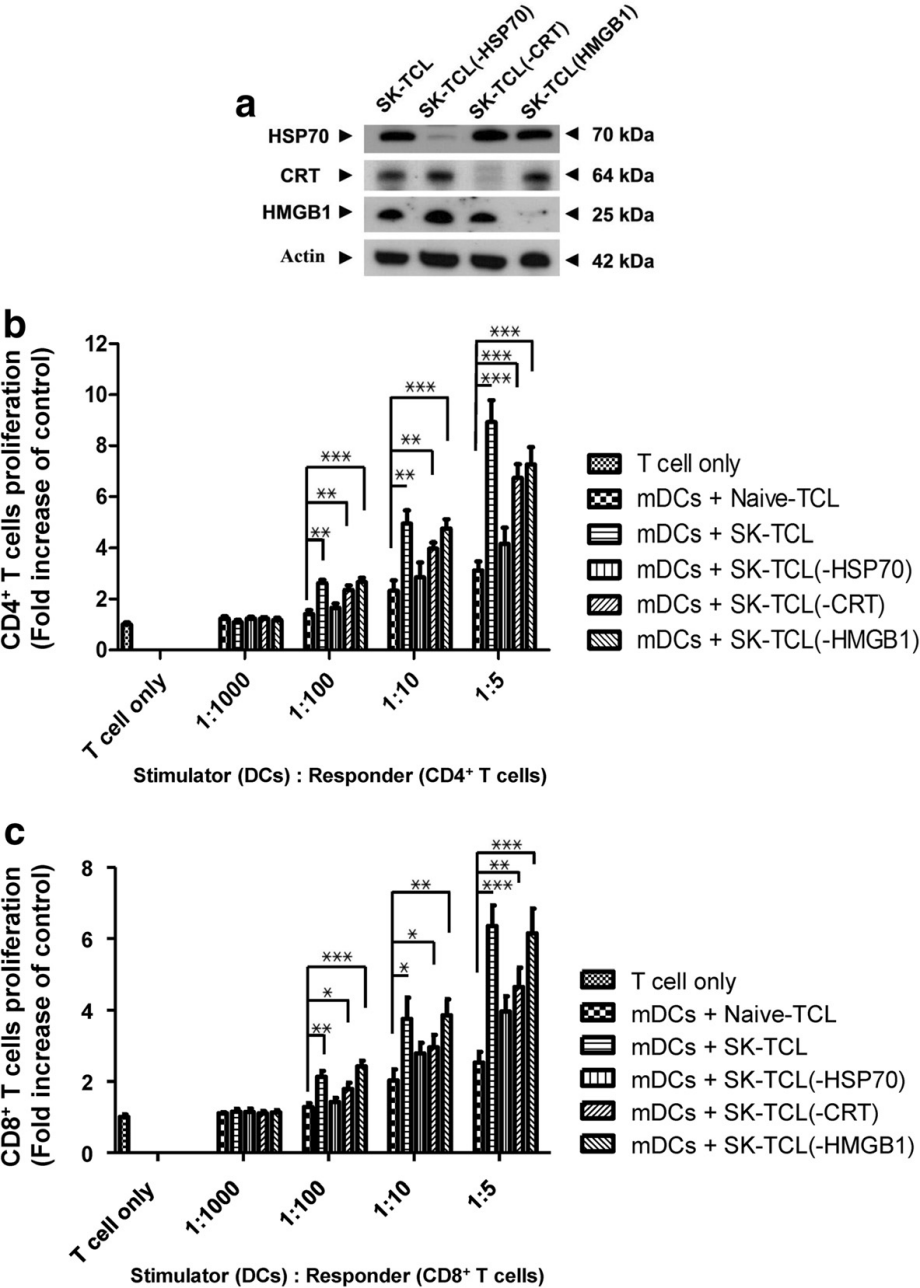
HSP70 and CRT may play an important role in shikonin-induced ICD-derived proteins in treated tumor cells, that may differentially activate DCs and mediate T-cell proliferation. The shikonin-treated 4 T1 cells and the resultant-ICD derived TCLs (i.e., SK-TCL) and the specific protein-deleted SK-TCL [(i.e., SK-TCL(−HSP70), SK-TCL(−CRT) or SK-TCL(−HMGB1)] were used to stimulate DCs and for subsequent activation of T-cell proliferation. (**a**) Western blot analysis of ICD component proteins in SK-TCL(−HSP70), SK-TCL(−CRT) and SK-TCL(−HMGB1) samples, representing specific depletion of HSP70, CRT or HMGB1 proteins in the correspondent SK-TCL sample preparations, respectively. These DC vaccine samples were then used as stimulator cells for T-cell proliferation assays. Splenic CD4^+^ and CD8^+^ T cells were collected from syngeneic mice as responder cells. Proliferation activities of (**b**) CD4^+^ and (**c**) CD8^+^ T cells were analyzed by MLR assay. Ratios of stimulator to responder cells were set between at 1:1000 and 1:5. T-cell proliferation activity is represented as the fold change over the control (T cells only). Data represent the mean ± SD of three replicates. Similar results were obtained from three independent experiments

To investigate the possible specific and compartmentalized effect of different ICD-related protein components (i.e., HSP70, CRT and HMGB1) in the SK-TCL complex on the activity of DC-activated T-cell proliferation**,** an antibody depletion experiment was performed. Test mDCs were stimulated with SK-TCL or the same complex in which a single specific ICD component protein (i.e., HSP70, CRT, or HMGB1) was effectively depleted *in vitro* by an antibody adsorption/pull down procedure. Western blot analysis (Fig. 4a) showed that the three HSP70-, CRT- and HMGB1-depleted SK-TCL samples were successfully obtained, and designated as SK-TCL(−HSP70), SK-TCL(−CRT) and SK-TCL(−HMGB1), respectively. The resultant DC-activated T-cell proliferation was then tested by MLR assay. Test results revealed that the splenic T-cell proliferative responses were dose dependently increased by co-cultivation of test mDCs, subjected to treatment with different TCL preparations [i.e., Naive-TCL, SK-TCL, SK-TCL(−HSP70), SK-TCL(−CRT) and SK-TCL(−HMGB1)] with test T-cells, at a ratio between 1:100 and 1:5 (DCs:T cells). At the ratio of 1:5, the SK-TCL, SK-TCL(−HSP70), SK-TCL(−CRT) and SK-TCL(−HMGB1) test samples showed an approximate 2.9-, 1.3-, 2.2- and 2.3-fold change in CD4^+^ T-cell proliferation, respectively, as compared to the control group (Naive-TCL) (Fig. 4b). In terms of CD8^+^ T-cell proliferation, activation by DCs with SK-TCL, SK-TCL(−HSP70), SK-TCL(−CRT) and SK-TCL(−HMGB1) samples showed an approximate 2.5-, 1.6-, 1.8- and 2.4-fold change in stimulatory activity, respectively, as compared with the Naive-TCL treated group (Fig. 4c). Among all tested SK-TCL samples, HSP70-depleted SK-TCL had the lowest stimulatory effect (i.e., 1.3-fold change) on CD4^+^ T-cell proliferation. In comparison, both the HSP70- and CRT-depleted SK-TCL samples had a similarly low level of effect (i.e., 1.6- and 1.8-fold change, respectively) on CD8^+^ T-cell proliferation. Therefore, these results suggest that HSP70 is the key component of SK-TCL complex that can mediate DC-activated CD4^+^ and CD8^+^ T-cell proliferation; CRT and HMGB-1 proteins seem to play a “supportive role” in this activity. In comparison, both HSP70 and CRT appear to play an equally important role in the proliferation of CD8^+^ T cells via activation of DCs by SK-TCL.

### HSP70, but not other ICD-related protein components, plays a key role in the suppression of metastatic 4 T1 malignancies.

**Fig. 5:**
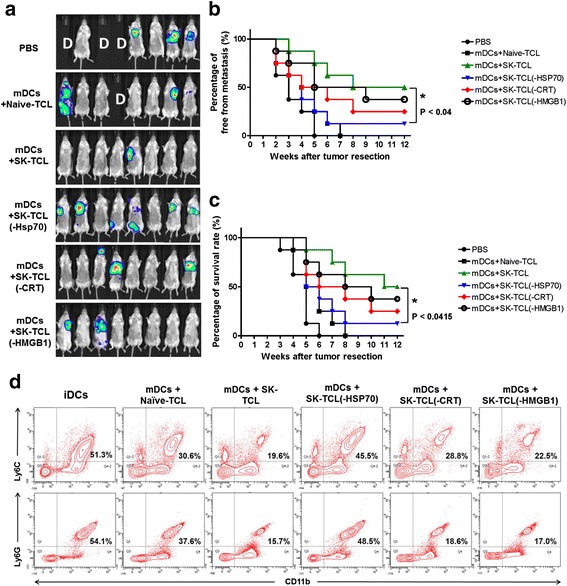
HSP70 may play a key role in the shikonin-induced ICD for enhancing anti-metastasis activity of DC-based cancer vaccines. Specific ICD protein components (i.e., HSP70, CRT or HMGB1) were depleted from SK-TCL samples before they were used as an adjuvant for preparation of a DC-based cancer vaccine. Test mice (8 mice/group) were injected with different vaccine formulations [i.e., iDCs, mDCs + Naive-TCL, mDCs + SK-TCL, mDCs + SK-TCL(−HSP70), mDCs + SK-TCL(−CRT), mDCs + SK-TCL(−HMGB1)] three times (see Materials and Methods), and mice were monitored for metastasis status and survival rate was scored for three months. **a** Representative *in vivo* bioluminescent images of test mice treated with different vaccine regimes after the resection of the 4 T1 orthotopic primary tumors. The red signals represent the highest level on the colorimetric scale. **b** Percentage of whole body organ free from metastasis in mice. The metastasis levels of tumors in test mice were scored by bioluminescence imaging within the indicated time period. **c** Survival rate of test mice treated with the indicated DC vaccine formulations, after tumor resection. A P value of less than 0.05 was considered significant (*, *P* < 0.05). **d** Ten days after the last vaccination, the percentages of monocytic MDSCs (CD11b^+^Ly6C^+^) and granulocytic MDSCs (CD11b^+^Ly6G^+^) in the blood of test mice were analyzed using flow cytometry. Similar trends of results were obtained from three independent experiments shown in (**a**), (**b**) and (**c**), and from two independent experiments shown in (**d**)

As seen in Fig. 4, HSP70 can play a key role in both CD4^+^ and CD8^+^ T-cell proliferation *in vitro*. Therefore, next we were interested in assessing whether HSP70 also serves as a major component of the SK-TCL complex that confers strong adjuvant activity for a DC-based cancer vaccine against metastasis of 4 T1 cells tested in a tumor resection mouse model. Bioluminescent imaging analysis of mice treated with control (PBS) and different vaccine adjuvant formulations [i.e., mDCs + Naive-TCL, mDCs + SK-TCL, mDCs + SK-TCL(−Hsp70), mDCs + SK-TCL(−CRT) and mDCs + SK-TCL(−HMGB1)] were scored and analyzed. As shown in Fig. 5a and 5b, among all the tested groups, tumor metastasis activity was strongly suppressed in mice of the mDCs + SK-TCL vaccinated group. However, when one specific ICD component protein (i.e., HSP70) was depleted, the SK-TCL(−Hsp70) vaccine was found to be inefficient in promoting the anti-4 T1 metastasis activity as compared to the mDCs + SK-TCL vaccine (Fig. 5a and 5b). At the third month post tumor resection, half of the mice of the mDC + SK-TCL vaccine group were free from tumor metastasis, in contrast, more than 85 % mice in mDC + SK-TCL(−HSP70) vaccine group suffered tumor metastasis (Fig. 5b). In addition, the survival time of test mice in the mDCs + SK-TCL(−Hsp70) group was found to be significantly decreased when compared to that of the mDC + SK-TCL group (Fig. 5c).

Myeloid-derived suppressor cells (MDSCs) are known to be an important component for regulation of tumor growth [15, 16]. The MDSC population consists mainly of two subsets: cells with granulocytic (gMDSC) phenotype expressing Ly6G marker, and cells with monocytic (mMDSC) phenotype expressing Ly6C marker [17]. Therefore, next we analyzed the possible effect of the test vaccines on inhibition of both granulocytic and monocytic MDSCs in immunized animals. The populations of monocytic and granulocytic MDSCs in peripheral blood of test mice were comparatively analyzed at the third week post tumor resection. As shown in Fig. 5d, compared with the mDCs + Naive-TCL group, the CD11b^+^Ly6C^+^ and CD11b^+^Ly6G^+^ MDSC populations in mice from the mDCs + SK-TCL group were significantly decreased, from 30.6 % to 19.6 %, and from 37.6 % to 15.7 %, respectively. When compared to the mDC + SK-TCL group, mice of the mDCs + SK-TCL(−HSP70) group exhibited a drastically increased cell population of CD11b^+^Ly6C^+^ and CD11b^+^Ly6G^+^ MDSCs, close to 45.5 % and 48.5 %, respectively (Fig. 5d). However, expansion of the monocytic and granulocytic MDSCs in mice that were treated with mDCs + SK-TCL(−CRT) or mDCs + SK-TCL(−HMGB1) exhibited only a 17.0 % and 28.8 % increase (Fig. 5d), respectively. These results suggest that HSP70 plays a highly significant, presumably major key role in SK-TCL-induced DC immunity, and is very effective for suppression of both CD11b^+^Ly6C^+^ monocytic and CD11b^+^Ly6G^+^ granulocytic MDSCs activities under *in vivo* vaccination conditions in mice.

### Both HSP70 and HMGB1 in SK-TCL are essential for activating the secretion of specific chemokines by treated DCs

**Fig. 6:**
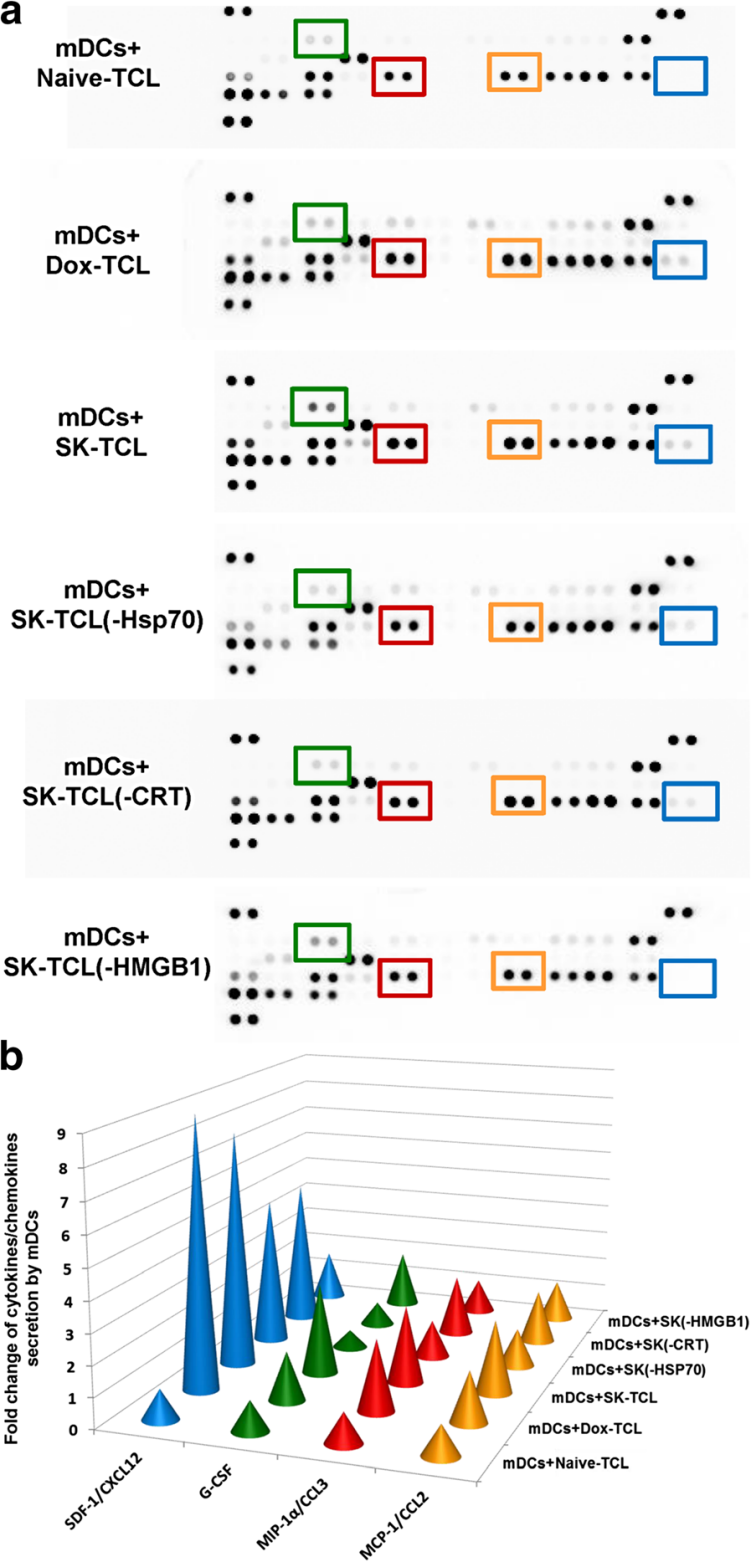
Cytokine profiling array analysis of conditioned culture media from various 4 T1 TCL-treated DCs. **a** Cytokine array membranes were incubated with cultured media from immature DCs or DCs that were treated with different TCL samples, including Naive-TCL, Dox-TCL, SK-TCL, SK-TCL(−Hsp70) , SK-TCL(−CRT) and SK-TCL(−HMGB1) for 2 h, and then stimulated with LPS for another 22 h. Squares mark the chemokines secreted from DCs that were increased in the SK-TCL-loaded group, as compared with those from Naive-TCL-loaded groups. Green square: G-CSF, blue square: SDF-1/CXCL12, red square: MIP-1α/CCL3 and yellow square: MCP-1/CCL2. **b** Stimulation (in fold change) of ICD-responsive chemokines in DCs loaded with Naive-TCL, Dox-TCL, SK-TCL, SK-TCL(−Hsp70), SK-TCL(−CRT) or SK-TCL(−HMGB1). Secretion level of each chemokine was normalized by value of Naive-TCL-loaded group (Fold change = Each TCL-loaded group/Naive-TCL-loaded group). Color of cone for each chemokine is correspondent to the indication in (**a**)

Previous studies have shown that specific spectra of cytokines and chemokines are needed to effectively mediate the innate and adoptive immune responses against tumor growth and metastasis, when mediated through the recruitment of DCs, monocytes, neutrophils, and T lymphocytes to the specific tumor sites (Dell'Agnola and Biragyn, 2007; Dranoff, 2004). Some cytokines and chemokines are known to play key roles in recruiting leukocytes or DCs into circulation and enhancing various cellular immune functions, including T-cell differentiation and polarization (Dranoff, 2004; Griffith et al., 2014). In order to dissect the functional, and potentially differential, effect of different ICD-associated molecules on cytokine/chemokine secretory activity in SK-TCL-loaded DCs, we analyzed the expression of a series of immunomodulatory cytokines and chemokines in the conditioned media of test DCs, using specific cytokine array analysis. As seen in Fig. 6a and 6b, SK-TCL-loaded DCs (mDCs + SK-TCL), as compared to Naive-TCL-loaded DCs (mDCs + Naive-TCL), expressed substantially higher activity in secreting a range of chemotaxis-related proteins, including SDF-1/CXCL12 (7.86-fold), G-CSF (3.02-fold), MIP-1α/CCL3 (2.57-fold) and MCP-1/CCL2 (2.41-fold). This result suggests that TCL collected from metastatic 4 T1 cells exhibiting SK-induced ICD activity can further enhance the secretory activity of specific chemokines from treated DCs. To evaluate the role of individual ICD-related protein components in this enhancement activity, an antibody-mediated protein pull-down experiment was conducted for depletion of three key ICD component proteins in SK-TCL samples. In comparison to other test vaccines, SK-TCL(−Hsp70)-loaded DCs [mDCs + SK-TCL(−Hsp70)] exhibited a significantly reduced level of G-CSF (from 3.02- to 0.60-fold), MIP-1α/CCL3 (from 2.57- to 1.24 fold) and MCP-1/CCL2 (from 2.41- to 1.20-fold), suggesting that HSP-70 has an important role in activating expression of these chemokines in test DCs. SK-TCL(−HMGB1)-loaded DCs [mDCs + SK-TCL(HMGB1)] showed a significantly decreased secretary SDF-1/CXCL12 (from 3.02- to 0.60-fold) and MIP-1α/CCL3 (from 2.57- to 1.24-fold) activity, suggesting that HMGB1 can enhance the expression of CXCL12 and CCL3 in test DCs. The SK-TCL(−CRT)-loaded DCs [mDCs + SK-TCL(CRT)] resulted only in reduced G-CSF expression (from 3.02- to 0.79-fold), as compared with SK-TCL-loaded DCs (Fig. 6a and 6b). Taken together, data from this cytokine/chemokine profiling experiment further indicate a distinguishable and differential functional effect of different ICD-component proteins on TCL-treated DCs, and these activities in combination may effectively augment the capacity of DCs to recruit other immune cells and polarize T cells, resulting in a potent vaccination effect.

### HSP70 and HMGB1 in SK-TCL are critical for activation of the expression of key DAMP-associated receptors on DCs

**Fig. 7:**
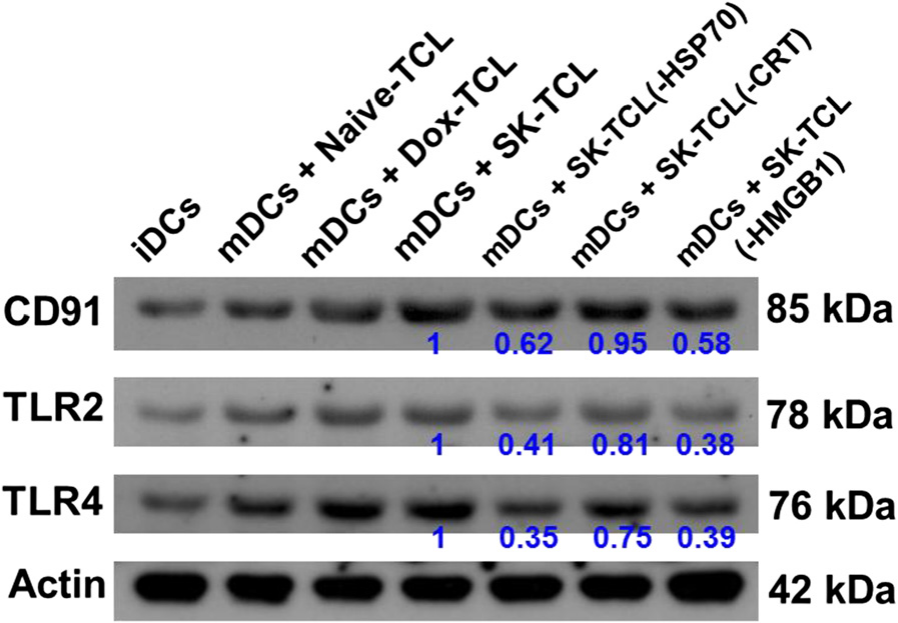
Western blot analyses on expression of CD91, TLR2 and TLR4 proteins on various TCL-activated DCs. Test 4 T1 cells were dispensed in 6-well plates (3 × 10^5^cells/well) and incubated with vehicle, SK or Dox at 5 μg/ml for 24 h. Some sets as replicates collected from SK-TCL samples were depleted for specific ICD molecules, i.e., HSP70, CRT or HMGB1, using an antibody-mediated pull-down procedure (see Materials and Methods). Different ICD molecule-depleted SK-TCL samples were then compared for their stimulatory activity on expression of CD91, TLR2 and TLR4 on TCL-pulsed DCs. Beta-actin was used as a loading control. The results represent the similar trend of data obtained from three independent experiments

Previous studies have shown that DAMP molecules, including the surface-exposed CRT, secreted ATP, HSP70 and HMGB1, and their interactions with the pattern-recognition receptors (PRRs), are required for presenting ICD activity, and this can ultimately lead to the activation of potent anticancer immunities [13, 18, 19]. In this study, we were able to detect specific changes in expression level/pattern of major DAMP-associated receptors on DCs, including CD91, TLR2 and TLR4, in response to priming with SK-TCL. In comparison, both Dox-TCL- and SK-TCL-pulsed DCs expressed much higher levels of expression of CD91, TLR2 and TLR4 than those detected in immature or Naive-TCL loaded DCs, suggesting specific ICD component proteins from treated tumor cells can stimulate the expression of these PRRs. By employing antibody-mediated depletion of specific ICD components, we further evaluated the role of each ICD component in stimulating PRR expression on the vaccinated DCs (Fig. 7). The SK-TCL(−HSP70) and SK-TCL(−HMGB1) vaccines showed significantly decreased stimulation of PRR expression on tested DCs. However, DCs activated by SK-TCL(−CRT) did not show a significant change in expression of PRRs (Fig. 7). These results suggest that both HSP70 and HMGB1 in SK-TCL are may play a critical role in activating the expression of key ICD molecules-associated receptors on DCs.

## Discussion

Recent studies on immune check point inhibitors, DAMP- and tumor associated antigen-mediated CTL activities, anti-MDSC activities, and DC-mediated tumor vaccine activities against metastatic tumors have generated renewed clinical interest in evaluating and optimizing cancer immunotherapy approaches. It is thus important to evaluate whether these various immune cell activities may be mechanistically correlated and clinically coordinated. The mechanism of export and uptake of DAMPs from immunogenic tumor cells to immature DCs remains unclear and deserves to be extensively explored to improve cancer vaccine formulation technology. The capacity of specific and individual DAMP components and their formulation as a complex in tumor cell lysate-activated DC vaccines to induce of the proliferation of CD4^+^ or CD8^+^ T cells, may play an important role in future design and development of anti-cancer vaccines, and hence deserves to be systematically studied for potential clinical application. In one of our previous studies, we demonstrated that shikonin can effectively induce the expression of specific DAMPs in treated tumor cells, and these DAMPs can in turn activate a cascade of caspase activities, which can, in combination, activate DCs to a full level of maturation and activation, resulting in potent Th17 and Th1 cell activities that mediate efficacious retardation of tumor growth and prolong the survival of test mice [8]. In this study, among various ICD-related protein components, both HSP70 and CRT were demonstrated to play an essential role in SK-TCL-induced DC immunity by stimulating both CD4^+^ and CD8^+^ T cell proliferation (Figs. 1 and 4). Our in vivo result is not in agreement, or perhaps even contradicts, reports that HMGB1 is the most vital determinant of the immunogenicity of ICD to DCs [20, 21]. Moreover, measurable differences was also observed in the effects of ICD-related protein components in tumor vaccine adjuvants against 4 T1 tumor metastasis and in mouse survival (Fig. 5a, b, c), i.e., HSP and CRT appear to play a primary and secondary role in these activities, respectively, and HMGB1 has limited or low effect.

Myeloid-derived suppressor cells (MDSC) are well known for their ability to suppress anti-tumor immune responses [16, 22]. Pre-clinical evidence suggests that cancer vaccines are more effective in tumor bearing mice that are depleted of MDSCs [23]. Our results, shown in Fig. 5d, suggest that HSP70 is a key or perhaps the only ICD component protein responsible for SK-TCL-induced alteration of MDSC activities, (including both Ly6G^+^CD11b^+^ granulocytic MDSCs and CD11b^+^Ly6C^+^ monocytic MDSCs) in test mice. To the best of our knowledge, the association between HSP70 expression and MDSC sub-population expansion has not been previously reported, and our present finding may warrant a future study on the possible cellular and molecular link(s) between MDSCs and specific ICD proteins, or HSP70 in particular, in the tumor tissue microenvironment, immunity, and the resultant tumor cell death.

Specific ICD proteins can interact with DAMP-associated receptors on DCs, such as DC91, TLR2 and TLR4, and promote the engulfment of dying cells [24]. TLR4 is the major receptor that recognizes the DAMPs exposed by tumor cells, which can facilitate intracellular antigen processing and presentation activities. TLR2, CD40 and CD91 are also known to mediate the binding and uptake of DAMP’s onto DC’s [25, 26]. Uptake of necrotic tumor cells can induce maturation of DCs to prime antigen-specific CD4^+^ and CD8^+^ T cells and the subsequent cellular immune responses [27]. Figure 7 shows that whereas both HSP70 and HMGB1 can play a key role in activating the expression of CD91, TLR2 and TLR4 on DCs treated *in vitro* with shikonin, much less effect (2–3 fold) was detected for the CRT protein. Correlation between the expression of HSP70 and HMGB1 and the subsequent rise in expressions of DAMP receptors has not been well characterized in previous studies, and our finding suggests that a positive feedback mechanism may exist, which can enhance DAMP receptor-mediated signaling activities in activated DCs.

Currently, the mechanism by which the key DAMPs direct DC migration and how the signals from these DAMPs are integrated and processed by the migrating dendritic cells is poorly understood. Our current study starts to reveal some of the possible component factors and their possible role in these highly integrated and likely programmed cellular and molecular activities. For instance, both HSP70 and CRT were demonstrated here to play a more essential role than HMGB1 on induction of ICD in test tumor cells. For future clinical considerations, we may spike in or supplement, individually or in combination, relatively high dose of recombinant human HSP70 or CRT protein preparations into patient’s tumor cell lysate, creating optimized ICD complex or cell lysate aggregates for vaccination. In addition, various patient tumor samples may also be treated ex-vivo in hospital laboratories with phytochemical shikonin or other similar adjuvant agents for enhanced expression of ICD proteins, effecting improved stimulation/activation of DC activities. Furthermore, various cellular and biochemical assays we used in this study may also be employed as “pre-vaccination diagnostic assays” for characterizing the patient’s tumor for modification or/and optimization of personalized cancer vaccines for specific individual cancer patients. With these considerations, we may thus hope to achieve an improved or enhanced anti-metastatic effect of future tumor vaccine “formulations”. This could especially be true if some of such measures could be practiced in combination with the exciting new immunotherapy strategy in using anti-PD1, −PDL1, and -CTLA4 antibodies. Taking together, future research is needed to design and manipulate phytochemicals or other treatment agents for the ex-vivo induction, engineering or reconstitution of custom made or re-constituted DAMPs that could be applied as a tailor-made combination or for specific individual patients, aiming to optimize to the utmost DC functions for trafficking and priming of T cells against metastatic tumor cells.

## Conclusions

Based on the results of the current study on the specific and functionally differential roles of three key ICD/DAMP proteins, mainly including HSP70, CRT and HMGB1 (Fig. 4, 5 and 6), in SK-induced ICD of tumor cells, we suggest that a new *ex vivo* approach may help rationally design and engineer some of the key component DAMPs proteins, in specific molecular ratios according to specific individual cancer patients’ needs, to supplement the TCL formulation from cancer patient’s tumors, i.e., delete, add or reconstitute TCL components to maximal effect, and thus to employ SK as an effective adjuvant in development of personalized dendritic cell-based anticancer vaccines.

### Ethics approval

We confirm that the animal experiments were approved by Institutional Animal Care and Use Committee (IACUC) of Academia Sinica ethics committee. The reference number is 12–01–304.

## Abbreviations

BMDC: Bone marrow-derived dendritic cells
CRT: Calreticulin
CTL: Cytotoxic T lymphocyte
DAMP: Damaged associated molecular pattern
DC: Dendritic cell
DOX: Doxorubicin
FBS: Fetal bovine serum
GM-CSF: Granulocyte-macrophage colony-stimulating factor
GRP: Glucose-related protein
HMGB1: High mobility group box 1
HSP: heat shock protein
iDC: immature dendritic cell
ICD: Immunogenic cell death
IVIS: *in vivo* imaging system
LPS: Lipopolysaccharides
mDC: mature dendritic cell
MDSC: Myeloid derived suppressor cell
MLR: Mixed lymphocyte reaction
PAMPs: Pathogen-associated molecular patterns
PBS: Phosphate buffered saline
PRRs: Pattern-recognition receptors
SK: Shikonin
TCL: Tumor cell lysates
TLR: Toll-like receptor
